# Replication of Reduced Pattern Electroretinogram Amplitudes in Depression With Improved Recording Parameters

**DOI:** 10.3389/fmed.2021.732222

**Published:** 2021-10-29

**Authors:** Evelyn B. N. Friedel, Ludger Tebartz van Elst, Céline Schmelz, Dieter Ebert, Simon Maier, Dominique Endres, Kimon Runge, Katharina Domschke, Emanuel Bubl, Jürgen Kornmeier, Michael Bach, Sven P. Heinrich, Kathrin Nickel

**Affiliations:** ^1^Department of Psychiatry and Psychotherapy, Medical Center—University of Freiburg, Faculty of Medicine, University of Freiburg, Freiburg, Germany; ^2^Eye Center, Medical Center—University of Freiburg, Faculty of Medicine, University of Freiburg, Freiburg, Germany; ^3^Faculty of Biology, University of Freiburg, Freiburg, Germany; ^4^Pfalzklinikum—Clinic for Psychiatry and Neurology, Klingenmünster, Germany; ^5^Center for Basics in Neuromodulation, Faculty of Medicine, University of Freiburg, Freiburg, Germany; ^6^Institute for Frontier Areas of Psychology and Mental Health, Freiburg, Germany

**Keywords:** pattern electroretinogram, PERG, depression, check size, dopamine

## Abstract

**Background:** The retina has gained increasing attention in non-ophthalmological research in recent years. The pattern electroretinogram (PERG), a method to evaluate retinal ganglion cell function, has been used to identify objective correlates of the essentially subjective state of depression. A reduction in the PERG contrast gain was demonstrated in patients with depression compared to healthy controls with normalization after remission. PERG responses are not only modulated by stimulus contrast, but also by check size and stimulation frequency. Therefore, the rationale was to evaluate potentially more feasible procedures for PERG recordings in daily diagnostics in psychiatry.

**Methods:** Twenty-four participants (12 patients with major depression (MDD) and 12 age- and sex-matched healthy controls) were examined in this pilot study. We investigated PERG amplitudes for two steady-state pattern reversal frequencies (12.5/18.75 rps) and four sizes of a checkerboard stimulus (0.8°, 1.6°, 3.2°, and 16°) to optimize the PERG recordings in MDD patients.

**Results:** Smaller PERG amplitudes in MDD patients were observed for all parameters, whereby the extent of the reduction appeared to be stimulus-specific. The most pronounced decline in the PERG of MDD patients was observed at the higher stimulation frequency and the finest pattern, whilst responses for the largest check size were less affected. Following the PERG ratio protocol for early glaucoma, where similar stimulus dependent modulations have been reported, we calculated PERG ratios (0.8°/16°) for all participants. At the higher frequency (18.75 rps), significantly reduced ratios were observed in MDD patients.

**Conclusion:** The “normalization” of the PERG responses—via building a ratio—appears to be a very promising approach with regard to the development of an objective biomarker of the depressive state, facilitating inter-individual assessments of PERG recordings in patients with psychiatric disorders.

## Introduction

As an ontogenetic part of the brain, the retina exhibits high levels of many neurotransmitters of the central nervous system, including dopamine (1). Since the retina represents a more accessible structure than the brain itself for related measurements, it recently gained increasing attention in other, non-ophthalmological research fields, such as neurology or psychiatry (2).

Indeed, previous studies indicate alterations in visual processing in diseases which are associated with a disturbance in the central dopaminergic homeostasis. These include Parkinson's disease (3–5), schizophrenia (6, 7) and major depressive disorder (MDD) (8–10).

Bubl et al. (8) initially reported higher contrast detection thresholds in patients suffering from MDD. In further research, they took advantage of a more objective electrophysiological approach from ophthalmology, the pattern electroretinogram (PERG), to demonstrate objective correlates of the essentially subjective state of depression (9).

The electroretinogram (ERG) uses corneal electrodes to measure the electrical activity of the retina in response to visual stimulation (11). The PERG is mostly generated by the retinal ganglion cells (12) which are stimulated by local contrast changes in black/white reversing pattern stimuli, like checkerboards (13, 14). The PERG allows both, an assessment of the macular function and a direct measurement of the retinal ganglion cell integrity (15). Therefore, it is—so far—primarily applied in ophthalmology for detecting early glaucomatous dysfunction (16–18).

In recent years, PERG has become increasingly important in psychiatric research as a possibility to map the integrity of the cerebral dopaminergic system indirectly via retinal ganglion cell function with minimal invasiveness (3, 19–22).

Bubl et al. (9), for instance, observed a remarkable reduction in the PERG contrast gain (corresponding to the increase in amplitude with ascending stimulus contrast) of about 50% in patients with MDD compared to healthy controls, with a significant negative correlation of contrast gain with depression severity. Moreover, with remission of the depressive symptoms, a normalization of the reduced retinal signals was observed (10). Therefore, it was postulated that the PERG could be a meaningful measurement tool for psychiatric disorders with the contrast gain as a state marker for depression (10). Table 1 lists preliminary and present investigations focusing on the contrast sensitivity and the PERG-based contrast gain in MDD patients.

**Table 1:** Previous studies on contrast sensitivity and/or PERG responses in patients with major depression.

**References**	***N* (Patients/HC)**	**Age (years) mean (SD)**	**Measurement parameters (e.g., pattern size, reversal rate, contrast level)**	**Results (Patients/HC)**
1. Bubl et al. (8)	28 MDD 21 HC	31.8 (9.5) 33.1 (10)	Gabor patches: Size: 2 cpd Contrasts: 1, 3, 10, 20, 30, 40, 50%	Elevated contrast discrimination threshold in MDD
2. Bubl et al. (9) [see technical note in Bubl et al. (23)]	40 MDD (20 medicated, 20 without medication) 40 HC	43.2 (6.3) 44.6 (4.5) 41.8 (4.5) 43.3 (6.3)	PERG Check size: 0.51° Reversal rate: 12.5 rps Contrasts: 3.2, 7.3, 16.2, 36, 80%	Reduced PERG contrast gain in MDD (~50% reduction in MDD)
3. Bubl et al. (10)	14 MDD (10 remitted, 4 not remitted) 40 HC	40.3 (12.8) 48.8 (9.8) 43.3 (12.7)	PERG Check size: 0.51° Reversal rate: 12.5 rps Contrasts: 3.2, 7.3, 16.2, 36, 80%	Normalization of reduced PERG-based contrast gain in MDD with remission
4. Fam et al. (24)	20 MDD 20 HC	44.5 (9.8) 43.7 (9.7)	(1) PERG Check size: 0.8° Reversal rate: 12 rps Contrasts: 7, 21, 42, 56, 68% (2) ffERG: flashes 0.01/3.0 cd·s/m^2^) (3) Contrast sensitivity (FrACT)	Normal signals in MDD for (1) PERG contrast gain and (2) ffERG; (3) Reduced contrast sensitivity in MDD which were correlated with BDI-symptoms

*BDI, beck depression inventory; cpd, cycles per degree; ffERG, full-field electroretinogram; HC, healthy controls; MDD, major depressive disorder; N, number; PERG, pattern electroretinogram; rps, reversals per second; SD, standard deviation*.

The International Society for Clinical Electrophysiology of Vision (ISCEV) published the current PERG standards in 2012 with recommendations for measurement parameters, calibrations and settings of PERG recordings (14). Various parameters have to be considered when recording the PERG signal.

For the standard PERG, a symmetrical black/white reversing checkerboard pattern with a constant mean luminance should be presented at a standard (15°) or large field size (≈ 30°). A check size of 0.8° (±0.2°), a reversal rate of approximately 16 rps (8 Hz) ±20% (for steady state stimulation) and a high stimulus contrast (> 80%) is recommended in the current guidelines (14). As the PERG amplitude increases almost linearly with increasing stimulus contrast (13), the PERG contrast gain can be calculated from a linear regression line (PERG contrast transfer function) as described by Bubl et al. (9). The slope of this regression line is modulated by the stimulus frequency as well as by the check sizes presented. Higher frequencies lead to a steeper slope of the PERG contrast transfer function, whereas the use of larger patterns (≈ 4°) seems to counteract this effect (25).

### Aims of the Study

The recommended standard recording parameters of the ISCEV (14) for clinical PERG assessment have been adapted for ophthalmologic patients. However, it has not yet been investigated whether they are equally suitable for PERG recordings in psychiatric patients.

The aim of the current study was (1) to replicate the findings of reduced PERG responses in patients with depression (9, 10) in an independent sample and (2) to improve the PERG protocols for this patient group with a specific focus on check size and stimulus frequency.

#### Check Size

It is known that the PERG amplitudes attenuate with poor visual acuity (26). Proper refraction is thus mandatory for PERG recordings, but difficult to implement in non-ophthalmological settings. This effect can be bypassed by using very coarse checkerboard patterns for the stimulation, which are clearly above the visual acuity threshold. In the present study, we investigated the PERG in response to a whole set of black/white reversal checkerboards with the following check sizes: 0.8°, 1.6°, 3.2°, and 16°.

#### Frequency

In previous studies about PERG effects in patients with depression, a stimulation frequency of 12.5 rps was applied. Higher reversal frequencies have been reported to be capable to increase the sensitivity for detecting ophthalmological diseases like glaucoma (27, 28). In the present study, we compared PERG amplitudes for two steady-state frequencies for pattern reversals (12.5 and 18.75 rps) to assess whether they can be applied equivalently.

## Materials and Methods

### Participants

The study was approved by the ethics committee of the University Medical Center Freiburg (Approval ID: 93/04) and was conducted in accordance with the Declaration of Helsinki. All participants gave their written informed consent. Patients were recruited at the Department of Psychiatry and Psychotherapy, University of Freiburg. The diagnosis of a major depressive episode was established by an experienced specialist in psychiatry according to DSM-5 criteria. A depressive episode in the context of bipolar disorder, the presence of psychotic symptoms, and comorbid alcohol abuse were defined as exclusion criteria. Initially, 17 patients with a diagnosis of major depressive disorder (MDD) were recruited. PERG measurement was performed within the first few weeks after starting antidepressant medication, without clinical response. The intake of neuroleptics, methylphenidate, or the antidepressant bupropion were defined as exclusion criteria.

In addition, 17 healthy controls without current or a history of psychiatric or neurological diseases were recruited. They had to score within the normal range of the Beck Depression Inventory [BDI; (29)] and the Hamilton Depression Rating Scale [HDRS; (30)]. The matching procedure controlled for effects of sex and age.

Exclusion criteria for both groups were defined as an age > 65 years, the presence of neurological or ophthalmological diseases or an uncorrectable low visual acuity (< 0.8).

The following questionnaires were collected from both patients and control participants: the Beck Depression Inventory [BDI; (29)] to assess the severity of depressive symptoms and the Wender-Utah Rating Scale [WURS-k; (31)] for ADHD symptoms in childhood. In addition, the Hamilton Depression Rating Scale [HDRS; (30)] was applied as third-party assessment questionnaire.

### Data Acquisition

Before examination, visual acuity of each participant was assessed monocularly with the Freiburg Visual Acuity and Contrast Test [FrACT; (32)] and, if necessary, corrected with refraction. A minimum of 0.8 decimal visual acuity was required for each eye.

DTL (Dawson, Trick, and Litzkow)-like electrodes (33), placed at the lower limbus of each eye were used for PERG recordings. Gold-cup electrodes positioned at each ipsilateral eye-canthus served as reference, an ear-clip as ground.

The EP2000 system was used for stimulation and initial data collection (https://michaelbach.de/sci/stim/ep2000/index.html; retrieval date 15.10.2021). Pattern stimuli were presented at an observer distance of 57 cm on a CRT monitor with 75 Hz frame rate in 800 x 600 pixel resolution, covering a field size of 32° × 27°. Symmetrical black/white reversal checkerboards with a mean luminance of 45 cd/m^2^ and a Michelson contrast of 80% served as pattern stimuli. Four different check sizes (0.8°, 1.6°, 3.2°, 16°) were presented using two different reversal frequencies (12.5 and 18.75 rps) in the steady state range. Every check size was presented for a duration of 10 sweeps with a constant sweep length for both frequencies (960 ms), starting with the lower reversal rate, followed by the higher one. Blocks for the different pattern sizes were shown in ascending manner (stepwise increasing check size: 0.8°, 1.6°, 3.4°, 16°). This sequence was repeated in 10 equal cycles, with a short break in between. Responses exceeding a threshold of 120 μV were automatically rejected as artifacts. A minimum of 100 artifact free sweeps were recorded per condition and submitted to stimulus-synchronized averaging.

### Data Analysis

First off-line data processing was performed in Igor Pro 7 (Wave Metrics) with the “EP2000” module. To eliminate mains hum artifacts, averaged response traces were digitally low pass filtered (40 Hz). A Fourier analysis was performed after any linear trend (e.g., due to baseline drifts) had been removed (34). PERG amplitudes were extracted from the Fourier spectra at the respective stimulation frequencies (12.5 and 18.75 Hz) and noise-corrected [as described in (34)]. The average magnitude from the direct adjacent frequencies served as noise estimate (35). Additionally, phases were extracted from the Fourier transformation.

### Statistical Analysis

Statistical analysis was carried out in “R” (36) with RStudio (37) using the “tidyverse” package (38) for data handling. For the 8 stimulus conditions (4 check sizes and 2 frequencies) PERG amplitudes from both eyes were averaged for every participant separately. Psychometric data comparisons and initial testing for differences between the groups or stimulation parameters were established using Wilcoxon rank sum tests [“rstatix” package (39)]. Response times (in ms) were calculated from the extracted phase values (40). The fully crossed factorial design was analyzed with a mixed analysis of variance (ANOVA) for repeated measures [“afex” package (41)]. The factors group, check size and stimulation frequency, as well as their interactions, were evaluated for their impact on PERG amplitudes or response times. The factors check size and frequency were considered as repeated measures factors for each subject. *Post-hoc* analysis was limited to group comparisons [“emmeans” package (42)] with equal variance assumed. Hedge corrected (43) Cohen's d was calculated as effect size estimation for unpaired samples [“rstatix” package (39)]. Significance levels were determined by applying the Bonferroni-Holm procedure for a familywise α of 0.05 (44).

## Results

### Demographic and Psychometric Data

Of the originally measured 17 patients, five had to be excluded. The reasons for exclusion were intolerance of the electrodes, the intake of neuroleptic medication, regular somatic medication, subsequently diagnosed psychiatric comorbidity and substance abuse. Finally, 12 patients between 19 and 51 years of age could be included in the final analysis. Four patients suffered from a first severe depressive episode, while 8 patients had a recurrent severe depressive episode. Of the 17 control participants measured, 12 were matched by sex and age to the included patients and considered in the final analysis. The psychometric data of the patient and the control groups are presented in Table 2.

**Table 2:** Demographic and psychometric data.

	**Characteristic**	**Controls, *N* = 12**	**Patients, *N* = 12**	***p*-value^a^**
Sex	Male	5/12 (42%)	5/12 (42%)	
	Female	7/12 (58%)	7/12 (58%)	
Age	Mean (SD)	29 (8)	26 (9)	0.087
	Range	20–51	19–51	
Medication	Medicated	0/12 (0%)	11/12 (92%)	
	Unmedicated	12/12 (100%)	1/12 (8.3%)	
WURS-k	Mean (SD)	11 (6)	23 (13)	0.003
	Median (IQR)	8 (7, 15)	18 (16, 27)	
BDI	Mean (SD)	3 (3)	25 (8)	<0.001
	Median (IQR)	3 (1, 5)	25 (18, 32)	
	(Missing)	0	1	
HDRS	Mean (SD)	1 (1)	22 (4)	<0.001
	Median (IQR)	1 (0, 1)	22 (20, 26)	

a *Statistical test: Wilcoxon rank-sum test. BDI, Beck Depression Inventory; HDRS, Hamilton Depression Rating Scale; IQR, interquartile range; N, number; SD, standard deviation; WURS-k, Wender-Utah Rating Scale; y, years*.

### Pattern Electroretinogram (PERG)

#### Group Averaged PERG Responses

In both groups, measures of one eye of each of two participants had to be excluded due to electrode displacement during the experiment. Except for these cases, the responses of both eyes were averaged before further analysis.

**Figure 1:**
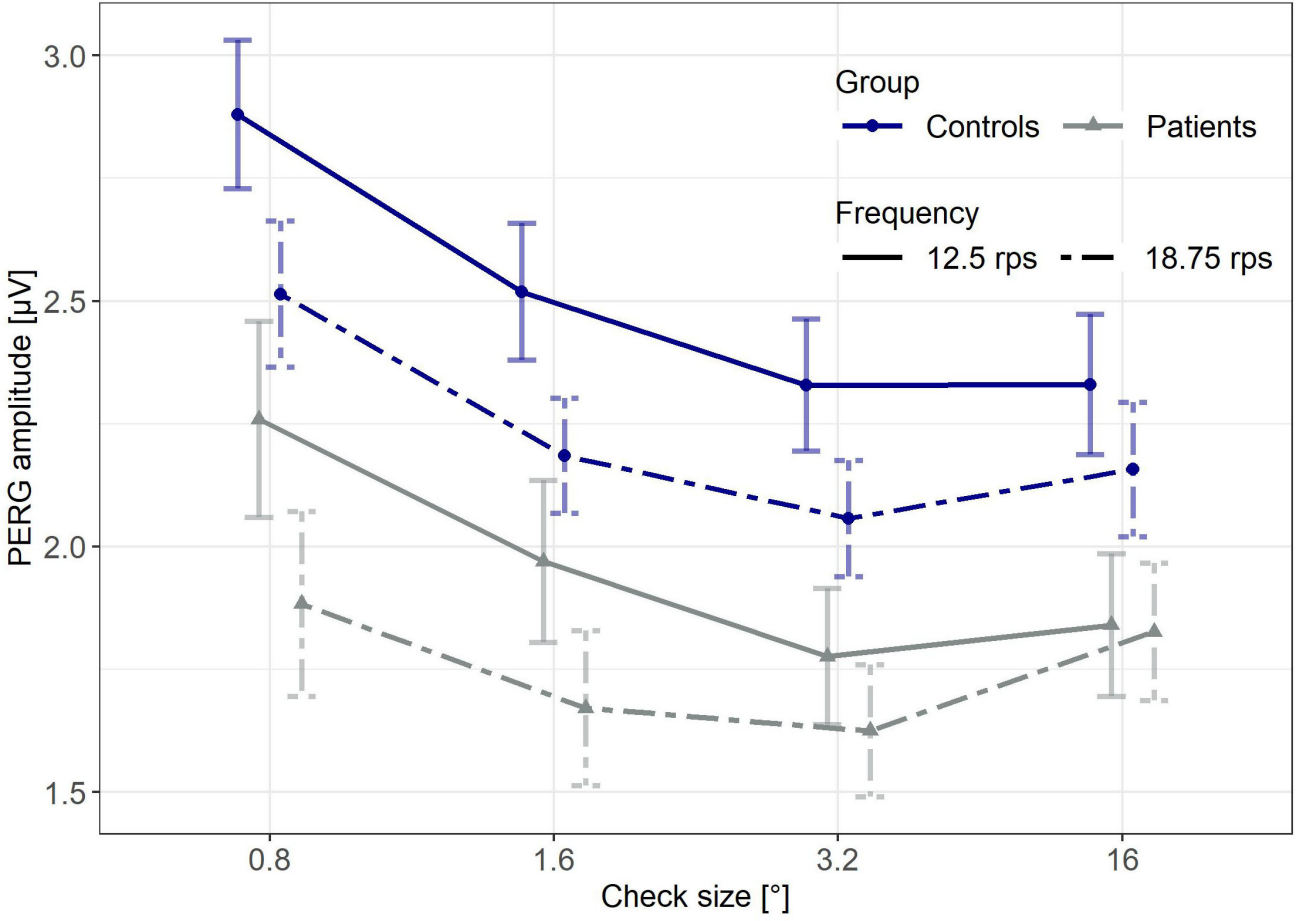
Mean PERG amplitudes for both groups and all stimulus conditions. Error bars indicate the standard error of the mean (SE).

Figure 1 illustrates the mean PERG amplitudes for patients and controls for all stimulus conditions.

The overall average of the PERG response of patients suffering from MDD was significantly lower compared to the control group (*p* = 0.017, unpaired, one-sided Wilcoxon test assuming lower PERG for MDD; data pooled across stimulus parameters), with a similar signal to noise ratio for both groups (*p* = 0.242, unpaired, two-sided Wilcoxon test, data averaged across stimulus parameters).

Although the average PERG amplitude in response to the higher frequency (18.75 rps) was significantly lower (*p* < 0.001; paired, one-sided Wilcoxon test assuming lower PERG at 18.75 rps; data averaged across groups and check sizes), the signal-to-noise ratio was comparable for both frequencies (*p* = 0.121; paired, two-sided Wilcoxon test, data pooled across groups and check sizes). Further visual inspection suggests largest amplitudes in response to the smallest check size with a slight attenuation toward coarser patterns.

The overall average of PERG response time was significantly reduced in patients with MDD compared to healthy controls (*p* < 0.001, unpaired, two-sided Wilcoxon test, data pooled across stimulus parameters).

#### Mixed ANOVA Results

With a mixed ANOVA, PERG amplitudes were evaluated for (between) group differences and influences from check size or stimulation frequency, both considered as subject-wise repeated measures (within). Possible interaction effects were included.

The ANOVA revealed a significant effect for the between factor group [*F*_(1, 22)_ = 6.53, *p* = 0.018] and the within factors check size [*F*_(1.52, 33.41)_ = 55.42, *p* < 0.001] and stimulation frequency [*F*_(1, 22)_ = 53.02, *p* < 0.001], as well as a significant interaction between the two stimulus parameters [*F*_(1.79, 39.38)_ = 19.24, *p* < 0.001] on the PERG amplitudes. Interactions between stimulus conditions and the factor group were not observed.

A separate ANOVA calculated for response times showed significant effects for the factors group [*F*_(1, 22)_ = 14.70, *p* < 0.001], frequency [*F*_(1, 22)_ = 88.14, *p* < 0.001] and size [*F*_(1.48, 32.51)_ = 1313.83, *p* < 0.001].

#### *Post-hoc* Analysis for Group Differences

A subsequent *post-hoc* comparison of the two groups indicated significantly reduced PERG amplitudes in the MDD group for almost all stimulus parameters (Table 3), considering the uncorrected results. After correcting significance levels for multiple comparisons according to the Bonferroni-Holm procedure, group differences remained significant only for the finest pattern (0.8°). At 18.75 rps, the decline of PERG in MDD (Table 3) apparently scales with the check size of the stimulus.

**Table 3:** Results from the *post-hoc* analysis for PERG amplitudes (in μV).

**Check Size [°]**	**Frequency [rps]**	**Controls mean (SD)**	**Patients mean (SD)**	**Differences of estimated marginal means (SE)**	***P*-value (significance level Holm adjusted)**	**Hedge corrected Cohen's d**
0.8	12.5	2.9 (0.5)	2.3 (0.7)	0.621 (0.21)	0.007 (^*^)	0.98
1.6	12.5	2.5 (0.5)	2 (0.6)	0.549 (0.21)	0.015 (ns)	1.00
3.2	12.5	2.3 (0.5)	1.8 (0.5)	0.553 (0.21)	0.014 (ns)	1.13
16	12.5	2.3 (0.5)	1.8 (0.5)	0.490 (0.21)	0.028 (ns)	0.95
0.8	18.75	2.5 (0.5)	1.9 (0.7)	0.630 (0.21)	0.006 (^*^)	1.04
1.6	18.75	2.2 (0.4)	1.7 (0.5)	0.514 (0.21)	0.022 (ns)	1.03
3.2	18.75	2.1 (0.4)	1.6 (0.5)	0.431 (0.21)	0.051 (ns)	0.95
16	18.75	2.2 (0.5)	1.8 (0.5)	0.331 (0.21)	0.127 (ns)	0.67

*Pairwise group comparisons (contrast: controls–patients) for all stimulus conditions with the corresponding differences in the estimated marginal means (SE included), the arithmetic mean and standard deviation (SD) for patients and controls as well as the effect size estimation (Cohen's d Hedge corrected) for group differences. Significance levels were adjusted according to Bonferroni-Holm procedure*.

* *significant; ns, not significant*.

In an additional *post-hoc* analysis, we detected shorter response times for patients with MDD compared to healthy controls for all stimulus parameter combinations (Table 4).

**Table 4:** Results from the *post-hoc* analysis for calculated response times (in ms).

**Check size [°]**	**Frequency [rps]**	**Controls mean (SD)**	**Patients mean (SD)**	**Differences of estimated marginal means (SE)**	***P*-value (significance level Holm adjusted)**	**Hedge corrected Cohen's d**
0.8	12.5	55.2 (2.9)	51.9 (3)	3.37 (0.96)	0.002 (^*^)	1.09
1.6	12.5	51.7 (2.6)	48.3 (2.8)	3.42 (0.96)	0.001 (^*^)	1.22
3.2	12.5	48.9 (2.2)	45.3 (2.4)	3.58 (0.96)	0.001 (^*^)	1.53
16	12.5	44.7 (2.1)	41.5 (2.4)	3.22 (0.96)	0.002 (^*^)	1.36
0.8	18.75	56.4 (1.8)	53.2 (2.5)	3.19 (0.96)	0.003 (^*^)	1.41
1.6	18.75	53.4 (2)	49.6 (2.3)	3.79 (0.96)	0.001 (^*^)	1.69
3.2	18.75	50.4 (2.1)	46.5 (2)	3.87 (0.96)	<0.0001 (^*^)	1.84
16	18.75	46.5 (2.1)	43 (1.8)	3.52 (0.96)	0.001 (^*^)	1.72

*Pairwise group comparisons (contrast: controls–patients) for all stimulus conditions with the corresponding differences in the estimated marginal means (SE included), the arithmetic mean and standard deviation (SD) for patients and controls as well as the effect size estimation (Cohen's d Hedge corrected) for group differences. Significance levels were adjusted according to Bonferroni-Holm procedure*.

* *significant; ns, not significant*.

#### Cohen's d Effect Size Estimation for Stimulus Parameter Combinations

**Figure 2:**
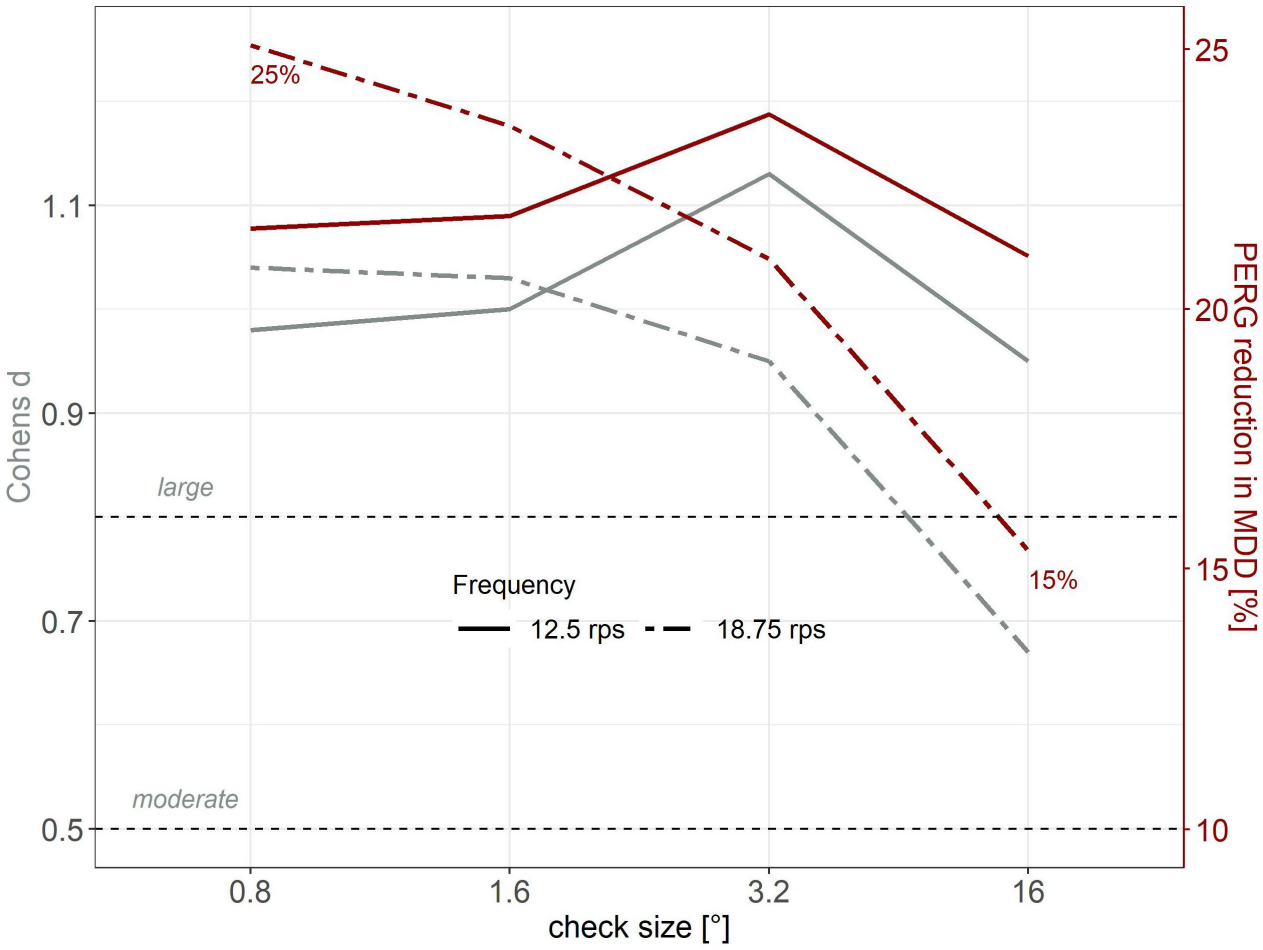
Cohen's d (gray, left axis) as effect size estimation and mean relative PERG reduction (red, right axis, %) in MDD patients compared to healthy controls as a function of check size. Continuous lines depict 12.5 rps, dashed lines represent 18.75 rps.

Figure 2 shows that, at a stimulation frequency of 18.75 rps, the PERG response difference between patients and healthy controls is gradually smaller with increasing check size. The most prominent decay in patients' PERG amplitudes was observed with the finest pattern (0.8°) (25%, *d* = 1.04), while the PERG amplitudes at the coarsest checkerboard (16°) seemed to be least affected (15%, *d* = 0.67). Interestingly, this PERG response pattern is reminiscent of the conditions observed in early glaucoma (16, 17, 45).

#### PERG Check Size Ratio

Based on the “PERG ratio protocol” in early glaucoma (17, 46, 47), check size ratios for the PERG amplitudes were established for both groups and frequencies according to formula (1).


(1) $$PERG\;ratio=\;\frac{PERG\;amplitude\;at\;0.8{}^{\circ}}{PERG\;amplitude\;at\;16{}^{\circ}}.$$

**Figure 3:**
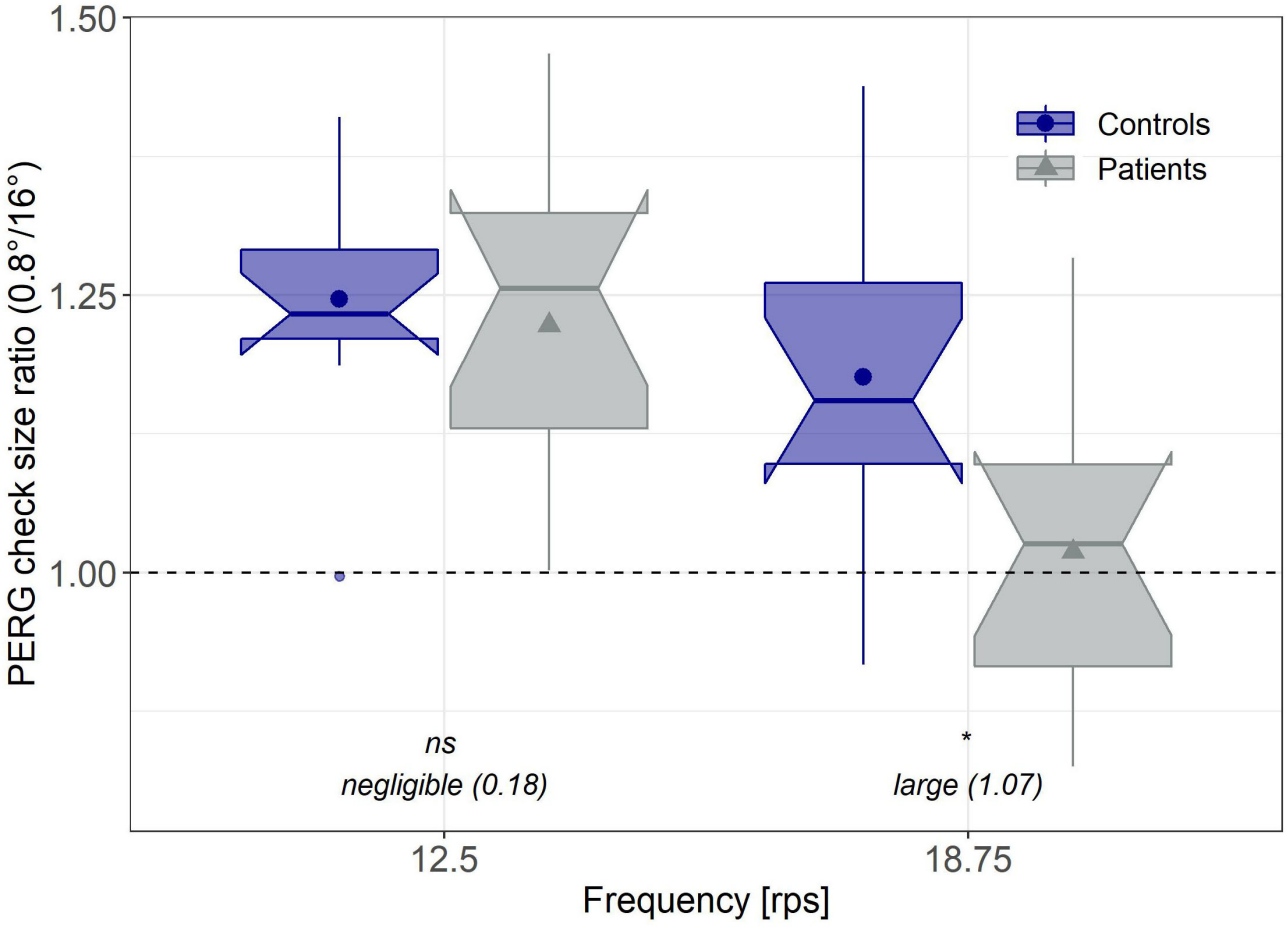
Normalized PERG amplitudes. Individual PERG ratios (0.8°/16°) corresponding to the “PERG ratio protocol” for early glaucoma (17) for both groups and frequencies. Significance levels were Bonferroni-Holm corrected. Effect sizes were estimated based on Hedge's corrected Cohen's d. *significant; ns, not significant.

With regard to the application of PERG response as an objective biomarker, the calculation of PERG ratios for every subject has the advantage of minimizing inter-individual variability by amplitude normalization. Figure 3 depicts the normalized PERG amplitudes for the patient and the control group.

A second mixed ANOVA with the between factor group, the within-factor stimulation frequency and the PERG ratio as dependent variable revealed a significant influence of stimulus frequency on the PERG ratio [*F*_(1, 22)_ = 29.23, *p* < 0.001], no overall-group differences [*F*_(1, 22)_ = 3.33, *p* = 0.082], but a significant interaction effect between group and stimulus frequency [*F*_(1, 22)_ = 6.96, *p* = 0.015].

*Post-hoc* evaluation exhibited that the PERG ratios in the MDD group, in response to a stimulation frequency of 18.75 rps, were significantly reduced (*p* = 0.008, *d* = 1.07), whereas with the lower reversal rate (12.5 rps), PERG ratios did not differ between groups (*p* = 0.674, *d* = 0.18).

## Discussion

The aims of the present study were (1) the replication of the PERG amplitude effect in patients with MDD and (2) the evaluation of different stimulus conditions to further improve PERG recording procedures for this patient group. Four check sizes (0.8°, 1.6°, 3.2°, and 16°) were compared to analyze if PERG signals in MDD, in response to coarser patterns, are affected to the same extent as to smaller check sizes used in previous studies. The application of coarser patterns would be beneficial by eliminating influences due to refraction errors. In addition, two frequencies (12.5 and 18.75 rps) for checkerboard reversals were investigated in order to test if PERG responses were similarly affected at higher stimulation frequencies.

### Group Comparisons for the Different Parameter Combinations

Overall, we discerned smaller PERG amplitudes in patients with MDD compared to matched healthy control participants, which replicates earlier findings with an independent sample of patients and controls (9). After correction for multiple testing, statistically reliable reductions in the PERG in MDD were only indicated with the smallest check size (0.8°).

This effect was not only present for the lower (12.5 rps), as previously reported (9, 10), but also for the higher stimulation frequency (18.75 rps) with a comparable signal-to-noise ratio for both frequencies. A follow-up study should address, whether a higher frequency can provide the opportunity to reduce total recording time. A shorter measurement time would be particularly advantageous for psychiatric patients with depressive symptoms and limited ability to uphold attention.

The higher rate for checkerboard reversals (18.75 rps) is additionally beneficial by fine-tuning group differences between MDD and control subjects through check size dependent modulations, which allow for a normalization of PERG responses via the calculation of check size ratios, similar to those used for the detection of early glaucoma (17).

Besides, our observations are not in line with the results of Fam et al. (24), who reported normal PERG contrast gain in MDD patients applying a stimulation frequency of 12 rps and the check size of 0.8°. This could possibly be due to differences in the technical implementation of the measurement protocol.

Since the PERG amplitude reduction in patients with MDD was only significant with the smallest check size (0.8°), we cannot recommend a recording paradigm which uses exclusively larger check sizes (1.6°, 3.2°, or 16°) in the context of psychiatric disorders, which would render the correction of refractive anomalies unnecessary.

Considering the dopamine-dependent regulation of the receptive field sizes in the retina (48) and the assumption of a disturbed dopamine homeostasis in MDD (49, 50), check size specific PERG alterations in MDD patients seem convincing. Particularly, dopamine is known for its modulatory role in the light adaptation of the retina, favoring daylight vision with high acuity, a mechanism provided by the decoupling of horizontal cells in the retina, thereby shrinking the antagonistic surround structures of the receptive fields (48). A disturbed dopamine homeostasis probably results in alterations in the PERG signals in response to different check sizes as it was similarly described for patients suffering from Parkinson's disease (4, 5).

Moreover, shortened response times were observed in patients with MDD for all stimulus conditions. As described in transient stimulations (51), the effect of a shorter response time with larger check sizes is observed in both groups. In patients with glaucoma, not only reduced amplitudes but also shorter response times have been similarly described by Bode and colleagues (40). At this point, the authors discuss an effect observed by Viswanathan et al. (52) that leads to a shortening of the P50 peak time when N95 is eliminated. Whether such a differential change between the N95 and the P50, which both contribute to the PERG signal, occurs in MDD and can explain the observed changes in the steady-state response time would need to be addressed in future studies.

### PERG Ratio

While it would have been useful to find strong effects of depression with the very large check sizes, which would have obviated refraction, one can turn the relative constancy of these amplitudes into our favor by using them for individual normalization. Inspired by the PERG ratio protocol in early glaucoma (16, 17, 45), we compared the standardized PERG amplitudes, i.e., the amplitude ratio over the two check sizes (0.8° and 16°), between groups for both stimulation frequencies. The advantage of this “PERG ratio” approach is that it reduces inter-individual variability. We observed a significantly reduced PERG ratio in MDD patients compared to healthy controls for the high stimulation frequency (18.75 rps), but not for the low stimulation frequency (12.5 rps). This alternative analysis approach is promising since it increases interpersonal comparability and statistical power, which is particularly important for an objective biomarker. Moreover, higher stimulation frequencies could possibly reduce the time required for recording, which should be addressed in a follow-up study.

### Methodological Issues and Limitations

The present study provides promising perspectives for the optimization of PERG recording procedures in psychiatric settings. However, some limitations have to be mentioned.

Due to the small number of patients, the results of the current study must be considered preliminary. Follow-up studies with larger samples sizes could yield further information about the adaptation of stimulation frequency and check size for PERG recordings in psychiatric patients. It should be noted, however, that the PERG ratio is feasible to minimize inter-individual differences.

At the time of measurement, patients had already been taking antidepressant medication for a few days or weeks. In a previous study by Bubl et al. (9), however, a reduction in contrast gain was detected in both medicated and un-medicated depressed patients. Another limitation is that smoking status was not considered as a matching factor between patients and controls. This could also have a confounding effect on results, as it could have an impact on dopamine neurotransmission (53). Lastly, the MDD group also exhibited elevated ADHD symptoms in childhood according to the WURS-k questionnaire compared to the control group. Since the PERG amplitudes from patients suffering from ADHD have been reported to be unaffected (54), we regard influences from ADHD symptoms as rather unlikely. Particularly since the so-called PERG noise, which has been shown to be elevated in ADHD patients (55), was not affected in our MDD patients (*p* = 0.94, one-sided Wilcoxon test comparing PERG noise between groups, data pooled across stimulus parameters).

### Summary

In summary, in this methodological pilot study we could reproduce earlier findings of reduced PERG amplitudes in patients with depression as a potentially objective biomarker signal of the essentially subjective state of depression. In addition, we were able to methodologically improve the recording procedure by demonstrating the suitability of a higher stimulation frequency for recordings along with the introduction of an interpersonal normalization approach for the PERG signals, which further enhances the sensitivity of the method.

## Data Availability Statement

The original contributions presented in the study are included in the article, further inquiries can be directed to the corresponding author/s.

## Ethics Statement

The studies involving human participants were reviewed and approved by the Ethics Committee of the University Medical Center Freiburg (Approval ID: 93/04). The patients/participants provided their written informed consent to participate in this study.

## Author Contributions

KN and EF wrote the paper. EF, KN, CS, and SM performed the data and statistical analysis. LTvE, KN, DEb, MB, and EF organized the study and created the study design. KN, DEb, and DEn recruited the patients and established the diagnosis. MB and SH supported the methodological and technical realization for the collection of the electrophysiological data. CS and EF performed the measurements. LTvE, KD, SM, DEb, DEn, KR, EB, MB, JK, and SH revised the manuscript critically focusing on clinical and statistical aspects. All authors were critically involved in the theoretical discussion, composition of the manuscript, and read and approved the final version of the manuscript.

## Funding

Part of the study was funded by the DFG (HE 3504/11-1 | TE 280/24-1). The article processing charge was funded by the Baden-Wuerttemberg Ministry of Science, Research and Art and the University of Freiburg in the funding programme Open Access Publishing.

## Conflict of Interest

LTvE Advisory boards, lectures, or travel grants within the last three years: Roche, Eli Lilly, Janssen-Cilag, Novartis, Shire, UCB, GSK, Servier, Janssen, and Cyberonics. KD member of the “Steering Committee Neurosciences,” Janssen Pharmaceuticals, Inc. The remaining authors declare that the research was conducted in the absence of any commercial or financial relationships that could be construed as a potential conflict of interest.

## Publisher's Note

All claims expressed in this article are solely those of the authors and do not necessarily represent those of their affiliated organizations, or those of the publisher, the editors and the reviewers. Any product that may be evaluated in this article, or claim that may be made by its manufacturer, is not guaranteed or endorsed by the publisher.
